# The Exhaustion Triangle: How Psychosocial Risks, Engagement, and Burnout Impact Workplace Well-Being

**DOI:** 10.3390/bs15040408

**Published:** 2025-03-23

**Authors:** Raquel Lara-Moreno, Adelaida Irene Ogallar-Blanco, Nancy Guzmán-Raya, María Luisa Vázquez-Pérez

**Affiliations:** 1 Grupo de Investigación Psicología de la Salud/Medicina Conductual (CTS-267), Facultad de Psicología, Universidad de Granada, Campus de Cartuja, s/n, 18071 Granada, Spain; mlvazquez@fundacionsafa.es; 2 Depto Psicología Evolutiva y de la Educación, Facultad de Educación, Economía y Tecnología de Ceuta, Universidad de Granada, C/Cortadura del Valle, s/n, 51005 Ceuta, Spain; 3 Depto Personalidad, Evaluación y Tratamiento Psicológico, Facultad de Psicología, Universidad de Granada, Campus de Cartuja, s/n, 18071 Granada, Spain; 4 Depto Sociología, Universidad de Granada (España), C. Rector López Argüeta, s/n, 18001 Granada, Spain; nguzmanr@ugr.es; 5 Depto Pedagogía y Psicología, Centro Universitario SAFA, Adscrito Universidad de Jaén, Avda. Cristo Rey, 17 Úbeda, 23400 Jaén, Spain

**Keywords:** psychosocial risk factors, occupational well-being, burnout, engagement, occupational health

## Abstract

Employee burnout levels have risen due to teleworking, increased job demands, and the lack of clear boundaries between personal and professional life. This study evaluated burnout levels, occupational health (through the presence or absence of psychosocial risk factors), engagement, and well-being/job satisfaction in a sample of employees aged over 18 from varying sociodemographic backgrounds. Additionally, we sought to explore the relationships among these variables and their influence on workplace well-being. The sample comprised 112 employees aged 18 to 65 (of both genders). The instruments used included the Burnout Syndrome Scale (Maslach Burnout Inventory—Student Survey -MBI-SS-), the DECORE multidimensional questionnaire, the Utrecht Work Engagement Scale (UWES), and the General Work Well-Being Questionnaire (qBLG). The results indicated that overall workplace well-being levels are high, while the presence of psychosocial risk factors and burnout levels are moderate to low. Most variables correlated with each other in the expected directions. Furthermore, job well-being was inversely predicted by cynicism and burnout and positively predicted by support, engagement, and control. This study highlights the importance of workplace well-being and occupational health. Our findings suggest the need for intervention programs that include strategies to motivate employees, improve the work environment, and enhance stress coping mechanisms, among other areas.

## 1. Introduction

In 2023, burnout levels had significantly increased compared to previous years, driven largely by telework conditions, rising job demands, and blurred boundaries between work and personal life (Costin et al., 2023; Eurofound, 2023). These changes were further exacerbated by the COVID-19 (Coronavirus Disease, 2019) pandemic, which intensified existing trends and transformed work practices, leading to the adoption of hybrid and remote work models. While these models initially seemed beneficial, they have introduced new challenges in managing employee well-being (Costin et al., 2023; Soto-Rubio et al., 2020). The lack of clear separation between work and personal life has contributed to heightened emotional exhaustion and work overload, two core components of burnout (Leiter & Maslach, 2024; Van Zoonen & Sivunen, 2021). Research indicates a strong correlation between burnout and negative affective responses (Khalkhali et al., 2024; Koutsimani et al., 2023).

As a result, workplace well-being has emerged as a critical focus within organizational psychology due to its direct impact on employee productivity, health, and overall quality of life. Studies show that promoting well-being at work enhances individual and collective performance and reduces absenteeism, turnover, and health-related costs (Lubbadeh, 2020). Psychosocial risk factors, engagement, and burnout are essential factors that significantly influence workers’ well-being (Bakker & Demerouti, 2017; Maslach & Leiter, 2016; Sinclair et al., 2024).

Psychosocial risk factors refer to conditions within the work environment that can lead to stress, such as excessive workload, lack of control over tasks, and poor interpersonal relationships. When these factors are not effectively managed, they can negatively impact employees’ physical and mental health, leading to symptoms such as anxiety, depression, and, ultimately, professional burnout (Demerouti et al., 2019; Leka & Nicholson, 2019; Leiter & Maslach, 2024). Burnout is a response to prolonged job stress, marked by decreased energy, emotional exhaustion, and a cynical attitude toward work, all of which diminish job performance and personal satisfaction (Leiter & Maslach, 2024; Maslach & Leiter, 2016). However, these symptoms are nonspecific and often overlap with other mental health disorders, making diagnosis challenging (Koutsimani et al., 2023; Parker & Tavella, 2021).

Conversely, engagement is a positive, fulfilling state of mind related to work, characterized by high energy levels, dedication, and absorption in daily tasks (Bakker & Demerouti, 2017). High engagement is linked to greater well-being at work, as engaged employees tend to experience higher satisfaction, better performance, and a lower likelihood of burnout (Sinclair et al., 2024). However, in environments where psychosocial factors are poorly managed, even highly engaged employees can suffer negative effects (Maung et al., 2023), highlighting the crucial role of a healthy work environment.

Understanding the interrelationship between psychosocial risk factors, engagement, and burnout is key to identifying the dynamics that influence workplace well-being. A work environment with high psychosocial risks can reduce engagement and increase the likelihood of burnout, ultimately impacting the health and performance of employees. Thus, effectively managing these factors is crucial to creating a work environment that promotes both well-being and productivity (Eurofound, 2023; Lubbadeh, 2020).

### Study Aims and Hypothesis

Considering the definitions provided and the potential relationships between variables in the workplace, the aims and hypotheses of the study are as follows:

**Aims** **1:** *The primary aim of this research is to assess burnout levels, occupational health (defined by the presence or absence of psychosocial risk factors), engagement, and well-being/job satisfaction within a sample of working adults from diverse demographic backgrounds.*

**Hypothesis 1** **(H1):** *We expect to observe high current burnout levels and moderate well-being, health, and engagement.*

**Aims** **2:** *A secondary aim of this research is to examine the relationships between the analyzed variables and the influence of occupational health (characterized by the absence of psychosocial risks), burnout syndrome, and engagement on occupational well-being.*

**Hypothesis 2** **(H2):** *We anticipate correlations among all variables, with inverse relationships between opposing factors and direct relationships between related ones. Specifically, greater occupational health and well-being—along with higher job satisfaction—are expected to correspond with lower burnout and increased engagement, and vice versa.*

**Hypothesis 3** **(H3):** *Additionally, we hypothesize that occupational well-being will be positively influenced by good occupational health (i.e., the absence of psychosocial risk factors), low burnout, and high engagement.*

## 2. Materials and Methods

### 2.1. Study Design and Procedure

This study adopted a cross-sectional correlational design.

For the purposes of this study, we first created a questionnaire using Google Forms, which included the self-report measures mentioned earlier. The questionnaire consisted of three sections. The first section collected sociodemographic data, provided basic information about the study, and contained a consent statement outlining the voluntary nature of participation, the confidentiality of participant responses, and the absence of liability for participants. The second section included questions about burnout and general job satisfaction, while the third section focused on occupational health and engagement.

After the questionnaire had been created, it was distributed through social media platforms (Facebook, WhatsApp, Instagram, and Email), inviting workers from various sectors to participate. Participants were required to be of legal age and currently employed under a valid contract, regardless of the company or sector. They completed the measures individually in a single session.

This study adheres to the Declaration of Helsinki and does not involve medical experimentation, so it was not subject to approval by a local bioethics committee.

### 2.2. Participants

A total of 112 individuals of both genders, aged between 18 and 65 years, participated in this study after providing informed consent (see Table 1 for sociodemographic details). The inclusion criteria were as follows: currently being employed and being over 18 years old. Consequently, unemployed individuals or those under 18 were excluded from the study. The majority (63.4%) reported working between 31 and 40 h per week, while 9.8% worked between 1 and 10 h per week. Most participants (91.1%) were employed in the service sector (tertiary sector).

A priori sample size calculation was performed using a free online tool, G*Power (Faul et al., 2009), with a power level of 90% and an α level of 0.05, based on previous and similar studies (Žlibinaitė & Yucel, 2024; Veljković et al., 2021). This analysis indicated that a sample size of 112 would be sufficient for the study. The adjustment to a 90% power level is statistically justifiable, as it achieves a suitable balance between Type I and Type II error rates, thereby reducing the likelihood of failing to identify true associations, and it is grounded in the understanding that it provides a reasonable probability of detecting significant effects, particularly in exploratory research contexts. Although a 95% power level is often preferred in statistical analyses, it may be less feasible within the constraints of time and resource limitations.

The response rate was 100%, as all participants who answered the survey completed it in full.

### 2.3. Measures

The measurement scales used in this study, along with their key characteristics and psychometric properties, were as follows:

- Maslach Burnout Inventory—Student Survey (MBI-SS)

Developed by Maslach and Jackson (1981) and adapted to Spanish by Schaufeli et al. (2002), this scale assesses burnout syndrome through three key dimensions: exhaustion, cynicism, and professional efficacy. It comprises 15 items, with 5 items measuring exhaustion, 4 measuring cynicism, and 6 measuring professional efficacy. Responses are rated on a Likert-type scale from 0 (never) to 6 (always). Total scores range from 0 to 6 for each subdimension. High scores in exhaustion and cynicism, combined with low scores in professional efficacy, are taken to indicate burnout. Scores for each dimension are calculated by summing the items within each dimension and dividing by the number of items. Participants were classified into three levels of burnout—mild (burnout in one domain), moderate (burnout in two domains), and severe (burnout in all three domains). This scale demonstrates high validity and reliability, with an overall α = 0.89 and subscale reliability ranging from 0.73 to 0.98.

- General Work Well-Being Questionnaire (qBLG in its Spanish acronym)

Created by Blanch et al. (2010), this scale consists of 55 items across 6 subscales: affect (10 items), competence (10 items), expectation (22 items), somatization (5 items), burnout (4 items), and alienation (4 items). Responses are rated on a Likert scale from 1 (never) to 5 (always) in this study. The affect, competence, and expectation scales assess basic well-being, while the somatization, burnout, and alienation scales address collateral effects. Scores are calculated by summing the items in each subscale, with overall scores divided into basic well-being and collateral effects. This scale shows high validity and reliability, with an overall α = 0.90 and subscale reliability ranging from 0.85 to 0.93.

- DECORE Multidimensional Questionnaire

The DECORE questionnaire, developed by Luceño et al. (2005), consists of 40 items rated on a five-point Likert scale (1 = strongly disagree to 5 = strongly agree), assessing workers’ perceptions of psychosocial risks. It includes four subscales: organizational support (12 items), rewards (11 items), control (9 items), and cognitive demands (8 items). Total scores range from 40 to 200, with higher scores indicating a higher perception of psychosocial risk factors. This questionnaire demonstrates high validity and reliability, with an overall α = 0.85 and subscale reliability ranging from 0.62 to 0.84.

- Utrecht Work Engagement Scale (UWES)

Developed by Schaufeli et al. (2002), the UWES consists of 17 items measuring 3 engagement dimensions: vigor (6 items), dedication (5 items), and absorption (6 items). Responses are rated on a Likert scale from 0 (never) to 6 (always). Scores for vigor, dedication, and absorption are obtained by summing the items within each dimension. This scale shows high validity and reliability, with an overall α = 0.94 and subscale reliability ranging from 0.79 to 0.88.

### 2.4. Statistical Methods

To conduct the analyses presented in the study, we first performed preliminary and exploratory analyses. This step was necessary to detect and, if required, correct any data entry errors, missing values, or outliers and verify the assumptions for parametric testing.

The normality test revealed that most variables did not follow a normal distribution (Kolmogorov–Smirnov test, *p* < 0.05). The Levene test confirmed the homogeneity of variances for most variables (*p* > 0.05), allowing us to proceed with parametric tests for statistical analyses.

In addition to descriptive analyses, we conducted Pearson’s r correlations and stepwise multiple regression analysis. The significance level for all tests was set at *p* < 0.05.

## 3. Results

The descriptive statistics for all the psychosocial variables are displayed in Table 2.

The results for the three dimensions of burnout (MBI-SS) show the following: the mean level of current professional efficacy is 4.25 (SD = 1.51), exhaustion is 2.24 (SD = 1.43), and cynicism is 1.32 (SD = 1.19). For well-being/job satisfaction, the basic well-being scale, which combines the affect, competence, and expectations scales, showed a mean score of 161.64 (SD = 25.83). In contrast, the collateral effects scale, which includes somatization, burnout, and alienation, has a mean of 19.54 (SD = 7.48), notably lower than the basic well-being scale.

Regarding engagement, the dimensions of vigor (22.19), absorption (20.56), and dedication (22.96) show moderate-to-high values, with results being broadly similar across these dimensions. Finally, the mean score for occupational health is 114.43 (SD = 20.44), indicating a moderately low presence of risk factors in the sample studied.

Next, Table 3 presents the correlations between all the psychosocial variables studied and well-being/job satisfaction. It is evident that basic well-being is inversely correlated with cynicism and burnout and directly correlated with occupational health, indicating the absence of risk factors. Additionally, the collateral effects scale is inversely correlated with engagement, while the dimensions of engagement (vigor, dedication, and absorption) are directly correlated. However, it is important to note that the collateral effects scale does not correlate with one dimension of burnout—professional efficacy. The remaining significant correlations are detailed in Table 3.

Finally, we analyzed the impact of the studied psychosocial variables on occupational well-being using stepwise multiple linear regression. The results showed that basic well-being was inversely predicted by cynicism (marginally significant) and burnout, while the absence of control and lack of support were direct predictors of basic well-being (see Table 4).

## 4. Discussion

The general objective of this research was to assess the levels of burnout, occupational health (presence or absence of psychosocial risk factors), engagement, and well-being/job satisfaction in a sample of working adults with various sociodemographic characteristics. It was hypothesized that burnout levels would be high while well-being, health, and engagement levels would be moderate. However, our results do not support the initial hypothesis. In our sample, burnout levels were not high, while levels of occupational health, absence of psychosocial risks, engagement, and well-being/job satisfaction were moderately high. This suggests that the current changes in work models are not having a significant negative impact on workers’ well-being.

We believe this could be due to the current “business-as-usual” situation, as organizations and employees have had time to adapt to new circumstances. They have also developed strategies to address risk factors introduced by the COVID-19 pandemic, such as hybrid and remote working models (Maung et al., 2023). Hybrid work, for instance, offers flexibility and improves work–life balance, helping to reduce stress and burnout (Krajčík et al., 2023). Additionally, many organizations have begun implementing psychological support measures and fostering more collaborative work environments, further contributing to employee well-being (Leka & Nicholson, 2019). Research also indicates that the experiences gained during the pandemic have strengthened workers’ resilience in facing workplace challenges. By managing stress and communicating effectively, employees are now better equipped to handle psychosocial risks in their work environments (Gemine et al., 2021; Lubbadeh, 2020). While these adaptations likely played a significant role in the positive results observed, it is important to recognize that individual factors such as resilience, coping strategies, and personality traits, may have also contributed substantially to the overall well-being seen in the sample (Alonso-Tapia et al., 2019; Karanika-Murray & Michaelides, 2021). For instance, individuals with higher resilience may have developed adaptive mechanisms that help mitigate the negative effects of psychosocial risks, regardless of the work model adaptations (Hartmann et al., 2020; Karanika-Murray & Michaelides, 2021). Similarly, personality traits like optimism or conscientiousness could influence how employees perceive and respond to workplace stressors, further impacting their overall health and job satisfaction (Alonso-Tapia et al., 2019; Pérez et al., 2021). Moreover, spiritual beliefs or practices (such as mindfulness) might have provided an additional layer of coping, helping individuals find meaning and strength in stressful situations (Goilean et al., 2020; Wnuk, 2018). This aligns with findings in healthcare settings, where research on hospice and palliative care workers suggests that, despite their continuous exposure to death, they do not experience burnout at the same rate as other healthcare professionals (Dahò, 2021; Harris, 2013). This discrepancy indicates that workplace structures, specific coping mechanisms, meaning-making processes, and professional philosophies may play a crucial role in mitigating emotional exhaustion. If workers in such emotionally intense environments manage to maintain lower levels of burnout, understanding which strategies or workplace structures contribute to this resilience could be highly relevant for developing better burnout prevention measures in other occupational settings. These intrinsic factors could play a pivotal role in explaining the well-being levels observed and should be explored in future studies to fully understand the dynamics at play in workplace well-being.

We also aimed to determine whether there was a relationship between the various variables studied. Our hypothesis posited that there would be a relationship among all the variables. According to our expectations, opposing variables would have an inverse relationship, while similar variables would show a positive correlation. Specifically, we expected that higher levels of occupational health and well-being/job satisfaction would correspond to lower levels of burnout and higher levels of engagement, and vice versa. Our findings support this hypothesis. Most variables and their dimensions correlate with one another, as expected. These findings align with those of Maslach and Leiter (2016), who describe an inverse relationship between burnout and engagement, existing on a continuum of workplace well-being. Burnout represents the negative end, while engagement represents the positive end. This continuum was confirmed in our study, with burnout dimensions—such as exhaustion and cynicism—negatively correlating with engagement and its dimensions. Moreover, prior studies have confirmed that burnout dimensions, such as exhaustion and cynicism, are negatively related to the components of engagement, such as dedication and vigor (Lubbadeh, 2020; Salanova et al., 2010; Schaufeli & Bakker, 2004). Given that excessive job demands without sufficient resources increase burnout (Bakker & Demerouti, 2017), understanding the coping mechanisms used by hospice workers—such as structured emotional support and team cohesion (Dahò, 2021; Harris, 2013)—could offer valuable insights into workplace well-being beyond healthcare settings.

Additionally, the relationship between burnout and occupational health can be observed through the associations between cynicism, exhaustion, and professional efficacy. Higher levels of cynicism and exhaustion correspond to lower levels of occupational health, defined as the absence of psychosocial risk factors. Conversely, higher levels of professional efficacy are associated with better occupational health, highlighting the relationship between burnout and occupational health. Specifically, higher levels of cynicism and burnout among employees lead to a decline in occupational health, defined as the absence of psychosocial risk factors (Leka & Nicholson, 2019; Jain et al., 2021). Cynicism, characterized by a negative attitude toward work and the organization, fosters a toxic work environment that amplifies stress and lowers job satisfaction (Leiter & Maslach, 2024). Conversely, when employees feel competent in their roles, they report lower levels of burnout and cynicism, leading to improved occupational health (Döbler et al., 2022; Lubbadeh, 2020). These dynamics underscore the importance of investing in skills development and resource allocation to enhance employee performance and promote overall well-being.

Greater well-being at work is strongly associated with better occupational health, confirming a positive relationship between the two variables. Higher levels of well-being predict higher levels of occupational health and vice versa. In contrast, occupational health correlates negatively with the collateral effects scale, indicating that when occupational health decreases, negative effects may spill over into other areas of life. Various studies have substantiated this relationship, emphasizing the importance of creating work environments that foster job satisfaction and promote employees’ physical and mental health (Demerouti et al., 2014; Sinclair et al., 2024). For instance, research by Bakker and Demerouti (2017) found that employees who experience high job satisfaction report fewer symptoms of burnout and greater engagement with their work. This supports the notion that higher baseline well-being is closely associated with better occupational health. Similarly, findings by Jain et al. (2021) suggest that job satisfaction not only enhances physical and mental health but also reduces the likelihood of experiencing negative collateral effects such as stress and burnout. Conversely, when well-being is low, there is a notable increase in negative outcomes, such as heightened stress, anxiety, and burnout. These adverse effects ultimately impair employees’ overall health and job performance (Döbler et al., 2022; Martínez-Díaz et al., 2023; Sonnentag & Fritz, 2015).

Regarding the impact of psychosocial variables on well-being, the two negative dimensions of burnout—cynicism and exhaustion—are shown to have a detrimental effect on workplace well-being. As levels of cynicism and exhaustion rise, a negative correlation with work well-being becomes evident, meaning employees experience reduced satisfaction and health in their work environment (Leiter & Maslach, 2024; Sinclair et al., 2024). On the other hand, engagement acts as a protective factor, along with social support and control. Research indicates that engagement, combined with social support and control, can help mitigate the adverse effects of burnout (Bakker & Demerouti, 2017; Shahwan et al., 2024; Sonnentag & Fritz, 2015). Social support provides employees with a network of resources to navigate stressful situations, while control refers to employees’ ability to influence their work environment. These factors foster a sense of autonomy and capability, enhancing workplace well-being (Döbler et al., 2022; Jain et al., 2021).

## 5. Study Strengths and Limitations

This study stands out for its comprehensive approach in analyzing the interaction between psychosocial risks, engagement, and well-being in influencing burnout. Additionally, the diversity of the sample, consisting of employees from various sociodemographic backgrounds, strengthens the representativeness and generalizability of the findings. Finally, its results have high practical relevance, as they can contribute to the development of interventions and workplace policies aimed at mitigating exhaustion and promoting healthier and more productive work environments.

Despite the results obtained, this study has certain limitations. The sample size (112 participants) is relatively small and should be expanded in future research to ensure more robust findings. Efforts should also be made to balance the number of participants across the various sociodemographic, personal, and work-related variables studied. Additionally, data collection relied on self-reporting via online surveys. While practical, this approach may have excluded workers with limited access to technology or those with less technological knowledge. Incorporating alternative techniques in future studies could allow for broader participation and provide a more accurate assessment of participants’ working conditions.

## 6. Future Directions

To build upon the current findings, future research should prioritize larger and more diverse sample sizes to enhance the reliability and generalizability of the results. Expanding the study across different industries and cultural contexts would provide a more nuanced understanding of how burnout manifests in various work environments. Additionally, longitudinal studies could help determine causal relationships between psychosocial risks, engagement, and well-being over time. Future studies should also incorporate a combination of qualitative and quantitative methods, such as in-depth interviews or observational studies, to complement self-reported data and provide a more comprehensive understanding of employees’ lived experiences. Furthermore, future research could investigate individual differences, such as resilience, personality traits, and coping strategies, to better understand their role in moderating the relationship between psychosocial risks and burnout, allowing for the development of more tailored interventions. Additionally, given the increasing prevalence of remote work and digitalization, it is essential to explore how these evolving work structures impact burnout, engagement, and overall well-being. Finally, exploring the effectiveness of specific workplace interventions designed to mitigate burnout—such as flexible work arrangements, mental health support programs, and leadership training—could yield practical recommendations for organizational policies aimed at promoting employee well-being.

## 7. Conclusions

In summary, our findings show that burnout—particularly the dimensions of exhaustion and cynicism—negatively impacts employee well-being. As these symptoms intensify, engagement decreases, emphasizing the importance of promoting a healthy work environment to improve employee engagement. Psychosocial factors, such as inadequate social support and lack of control, if poorly managed, can exacerbate the negative effects of burnout. Conversely, fostering a supportive and communicative workplace can mitigate these effects and improve occupational health. In this regard, engagement emerges as a critical protective factor against burnout. Engaged employees report higher levels of satisfaction and well-being, and professional efficacy is positively associated with occupational health. These findings suggest that developing the coping skills of employees and providing necessary resources are key to promoting workplace well-being. Therefore, organizations should implement strategies that enhance well-being, such as social support initiatives and programs that increase employee autonomy and control. Such measures could reduce burnout, boost engagement, and improve occupational health. Moreover, organizations should consider these relationships to create healthier and more productive work environments. For instance, it may be possible to enhance employee well-being and prevent burnout by managing psychosocial factors and encouraging engagement. Finally, implementing organizational strategies focused on employee well-being not only benefits employees but also positively impacts productivity and the workplace culture.

## Figures and Tables

**Table 1:** Sociodemographic data of the sample.

Variable		N	%
Gender	Man	49	43.7
	Woman	63	56.3
Age	18 to 20 years	8	7.2
	21 to 30 years old	41	36.6
	31 to 40 years old	14	12.5
	41 to 50 years	22	19.6
	More than 50 years	27	24.1
Marital status	Single	31	27.7
	Married	48	42.9
	Divorced	8	7.1
	Widowed	0	0
	Stable couple	25	22.3
Income level (monthly)	Less than EUR 1000	37	33
	Between EUR 1000 and EUR 2000.	54	48.2
	Between EUR 2000 and EUR 3000.	19	17
	More than EUR 3000.	2	1.8
Workday	1–10 h per week	11	9.8
	11–20 h per week	15	13.4
	21–30 h per week	15	13.4
	31–40 h per week	71	63.4
Educational level	No education	2	1.8
	Primary	8	7.1
	Secondary	47	42
	University students	39	34.8
	Master’s/Doctorate	16	14.3
Company Type	Public	45	40.2
	Private	62	55.4
	Mixed	5	4.4
Size of company (no. of employees)	Microenterprise	23	20.5
	Small business	19	17
	Medium-sized company	47	42
	Large company	23	20.5
Sector *	Primary	2	1.8
	Secondary	8	7.1
	Tertiary	102	91.1

* The labor market is divided into three sectors: the primary sector (resource extraction), the secondary sector (manufacturing and construction), and the tertiary sector (service-based jobs like healthcare, education, and sales).

**Table 2:** Descriptive results for all psychosocial variables measured.

Variables	Min	Max	Mean	SD
Professional efficacy	0	6	4.25	1.51
Exhaustion	0	6	2.24	1.43
Cynicism	0	6	1.32	1.19
Affect Scale	10	70	37.41	6.70
Competencies	10	70	40.14	7.44
Expectations	21	147	94.09	13.47
Somatization	1	7	2.00	1.23
Burnout	3	15	8.28	3.32
Alienation	4	28	9.27	3.90
Well-being	41	287	161.64	25.83
Collateral Effects	8	56	19.54	7.48
Vigor	0	30	22.19	6.56
Absorption	0	36	20.56	6.67
Dedication	0	30	22.96	9.27
Engagement	0	96	65.71	19.01
Occupational health	40	200	114.43	20.44
Control	8	40	23.19	5.85
Support	12	60	41.95	7.92
Rewards	11	55	21.12	7.02
Cognitive Demands	9	45	21.04	7.97

**Table 3:** Correlations between the measured psychosocial variables (*p* < 0.05 *; *p* < 0.01 **).

	1	2	3	4	5	6	7	8	9	10	11	12	13	14	15
1 Prof. Efficacy	-														
2 Exhaustion	0.35 **	-													
3 Cynicism	0.25 **	0.63 **	-												
4 Affect		−0.47 **	−0.50 **	-											
5 Competencies		−0.38 **	−0.41 **	0.81 **	-										
6 Expectations		−0.45 **	−0.46 **	0.77 **	0.83 **	-									
7 Burnout		0.63 **	0.39 **	−0.35 **	−0.27 **	−0.33 **	-								
8 Alienation		0.51 **	0.45 **	−0.45 **	−0.42 **	−0.44 **	0.72 **	-							
9 Somatization	−0.25 **	0.25 **	0.20 *		−0.22 *		0.59 **	0.48 **	-						
10 Well-being.		−0.47 **	−0.49 **	0.89 **	0.93 **	0.96 **	−0.34 **	−0.47 **	−0.19 *	-					
11 Collat-effects		0.59 **	0.44 **	−0.42 **	−0.37 **	−0.40 **	0.92 **	0.92 **	0.68 **	−0.43 **	-				
12 Vigor	0.31 **		−0.25 **	0.34 **	−0.35 **	0.44 **				0.42 **		-			
13 Absorption	0.24 *				0.22 *	0.29 **				0.25 **		0.74 **	-		
14 Dedication	0.42 **		−0.20 *	0.34 **	0.36 **	0.47 **				0.44 **		0.88 **	0.75 **	-	
15 Occup-Health.		−0.20 *	−0.26 **	0.20 *	0.28 **	0.34 **		−0.21 *		0.31 **	−0.21 *				-
16 Control		−0.29 **	−0.26 **	0.34 **	0.40 **	0.41 **	−0.30 **	−0.42 **	−0.22 **	0.42 **	−0.39 **				0.76 **
17 Support	0.30 **		−0.30 **	0.35 **	0.40 **	0.47 **	−0.25 **	−0.39 **	−0.21 **	0.45 **	−0.35 **	0.24 *	0.26 **	0.24 *	0.81 **
18 Rewards		−0.27 **	−0.24 **	0.20 *	0.30 **	0.32 **	−0.29 **	−0.26 **	−0.24 **	0.30 **	−0.30 **				0.71 **
19 Cog-demands.															0.38 **

**Table 4:** Significant predictors of well-being at work.

	Predictors	*R* ^2^ *_cor._*	*β_stand._*	*t*	*p*
Basic well-being (F = 19.558, *p* = 0.000 **)	Cynicism	0.234	−0.173	−1.837	0.069 ^+^
	DECORE Support	0.329	0.197	2.244	0.027 *
	Engagement	0.394	0.293	4.040	0.000 **
	Exhaustion	0.439	−0.246	−2.666	0.000 *
	DECORE_Control	0.455	0.176	2.035	0.044 *

*p* < 0.1 ^+^; *p* < 0.05 *; *p* < 0.01 **.

## Data Availability

The data that support the findings of this study are not publicly available. The data are, however, available from the authors upon reasonable request.
